# Revisiting Plus-Strand DNA Synthesis in Retroviruses and Long Terminal Repeat Retrotransposons: Dynamics of Enzyme: Substrate Interactions

**DOI:** 10.3390/v1030657

**Published:** 2009-11-04

**Authors:** Daniele Fabris, John P. Marino, Stuart F.J. Le Grice

**Affiliations:** 1 Department of Chemistry and Biochemistry, University of Maryland Baltimore County, 1000 Hilltop Circle, Baltimore, MD 21228, USA; 2 Center for Advanced Research in Biotechnology of the University of Maryland Biotechnology Institute and the National Institute of Standards and Technology, 9600 Gudelsky Drive, Rockville, MD 20850, USA; 3 HIV Drug Resistance Program, NCI, National Institutes of Health, Frederick, MD 21702-1201, USA

**Keywords:** retroviruses, LTR-retrotransposons, polypurine tract, plus strand DNA synthesis, NMR spectroscopy, single molecule spectroscopy, antiviral strategies

## Abstract

Although polypurine tract (PPT)-primed initiation of plus-strand DNA synthesis in retroviruses and LTR-containing retrotransposons can be accurately duplicated, the molecular details underlying this concerted series of events remain largely unknown. Importantly, the PPT 3′ terminus must be accommodated by ribonuclease H (RNase H) and DNA polymerase catalytic centers situated at either terminus of the cognate reverse transcriptase (RT), and in the case of the HIV-1 enzyme, ∼70Å apart. Communication between RT and the RNA/DNA hybrid therefore appears necessary to promote these events. The crystal structure of the HIV-1 RT/PPT complex, while informative, positions the RNase H active site several bases pairs from the PPT/U3 junction, and thus provides limited information on cleavage specificity. To fill the gap between biochemical and crystallographic approaches, we review a multidisciplinary approach combining chemical probing, mass spectrometry, NMR spectroscopy and single molecule spectroscopy. Our studies also indicate that nonnucleoside RT inhibitors affect enzyme orientation, suggesting initiation of plus-strand DNA synthesis as a potential therapeutic target.

## 1. Introduction

Plus strand, DNA-dependent DNA synthesis in retroviruses and long-terminal repeat (LTR)-containing retrotransposons proceeds from the 3′ terminus of a purine-rich, RNA primer designated the polypurine tract (PPT) [1]. In contrast to genomic RNA of the RNA/DNA replication intermediate, PPT-containing RNA/DNA hybrids are refractory to degradation by the ribonuclease H (RNase H) activity of the cognate reverse transcriptase (RT). Once primer function has been satisfied, precise excision of the PPT from nascent DNA ensures that the 5′ terminal sequence of the double-stranded DNA provirus is recognized by the viral integration machinery. Given the importance of the PPT at different steps in the retroviral replication cycle, the absence of a common consensus sequence is somewhat surprising. This is perhaps most pronounced with PPTs of the *Saccharomyces cerevisiae* LTR transposons Ty1 and Ty3, which are simply a short run of alternating purines [1]. The all-purine nature of the PPT is also not inviolable, evidenced by a single pyrimidine in plus-strand primers of human immunodeficiency virus (HIV) type 2 (HIV-2) and Mason-Pfizer monkey virus [1]. Notwithstanding these divergent sequences, and possible differences in the structure of their cognate reverse transcriptase (RT), it would not seem unreasonable to assume that a common molecular mechanism underlies PPT recognition and precise elimination from plus-strand DNA.

**Figure 1.**
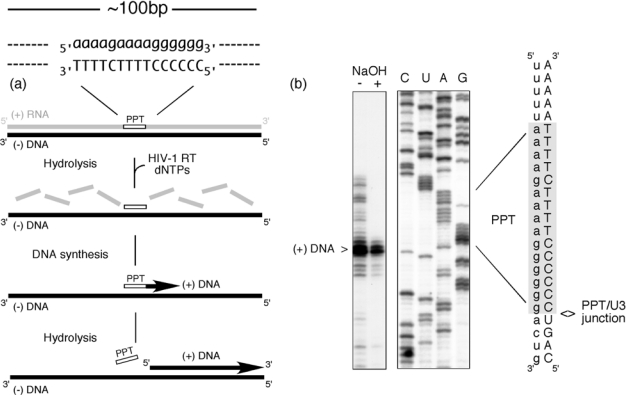
PPT-primed synthesis of HIV plus-strand DNA. The experimental strategy is outlined in (a), and comprises an RNA/DNA hybrid within which the PPT sequence is embedded. RNA and DNA nucleotides are in lower and upper case, respectively. Inclusion of HIV-1 RT and a full complement of dNTPs (one of which is radiolabeled) is predicted to support cleavage of the plus strand RNA (excluding the PPT) (RNase H-mediated), initiation of DNA synthesis from the PPT 3′ OH (DNA polymerase-mediated) and precise post-priming cleavage of the PPT at the PPT/U3 junction (RNase H-mediated). The experimental outcome is illustrated in (b). NaOH + and − notations indicate whether the final reverse transcription product was subjected to alkaline hydrolysis. C, U, A,G; sequencing reactions to locate the site of plus-strand initiation. Adapted from [2].

Despite the complexity of PPT utilization, Figure 1 illustrates that this multi-step process can be faithfully accomplished in the absence of accessory viral or host factors. In this experiment, a ∼100bp RNA/DNA hybrid, within which the PPT was embedded, was incubated with HIV-1 RT in the presence of dNTPs, one of which was radiolabeled [2]. The presence of a correctly-sized plus strand DNA product reflects a concerted series of steps whereby (i) RNase H activity creates the PPT primer 3′ OH (ii) DNA-dependent DNA polymerase activity initiates plus strand DNA synthesis from the liberated primer terminus and (iii) RNase H activity thereafter cleaves at the PPT/U3 RNA-DNA junction to release the primer. Since the PPT was embedded within a larger RNA/DNA hybrid, data in Figure 1 also implies that its recognition can be uncoupled from minus-strand DNA synthesis over the plus-strand RNA genome. Stated differently, early studies provided evidence for the importance of molecular cross-talk between HIV-1 RT and the PPT-containing RNA/DNA hybrid [1]. Another feature of plus strand DNA synthesis may involve co-evolution of the PPT sequence with its cognate RT, as suggested by our work on PPT usage by the RTs of HIV-1 (5′-a-a-a-a-g-a-a-a-a-g-g-g-g-g-g-3′) and the *S.cerevisiae* LTR retrotransposon Ty3 (5′–g-a-g-a-g-a-g-a-g-g-a-a-g-a-3′) [3]. In contrast to accurate selection of their cognate primer, Ty3 RT cleaves the HIV-1 PPT immediately downstream of the PPT/U3 junction, while HIV-1 RT non-specifically hydrolyzes the Ty3 PPT.

The availability of a high resolution structure of an RT/PPT complex would contribute significantly to how the primer terminus is recognized by catalytic centers located at the N- (DNA polymerase) and C-terminus of RT (RNase H) and, in the case of the HIV-1 p66/p51 heterodimer, almost 70Å apart [4,5]. Although Sarafianos *et al.* [6] have obtained a structure of HIV-1 RT with a PPT-containing RNA/DNA hybrid at 3.0Å resolution, the orientation the enzyme adopted positioned its RNase H catalytic site several base pairs from the PPT/U3 junction. We summarize here a multi-disciplinary approach designed to examine PPT structure and function that combines recently-developed chemical footprinting techniques, high-resolution mass spectrometry, solution-state NMR spectroscopy and single molecule spectroscopy to examine structural contributions from both the DNA and RNA strands of the RNA/DNA hybrid. Collectively, these state-of-the-art technologies illustrate that the “molecular gymnastics” of PPT utilization require extensive cross-talk between the protein and nucleic acid components, as well as long-range communication along the hybrid. A more detailed description of the biochemistry of PPT recognition, including (i), site-directed mutagenesis to investigate the contribution of individual nucleotides (ii) programmed initiation of plus-strand DNA synthesis and (iii) its cessation at the central PPT/central termination sequence, can be found in [1] and references therein.

## 2. Chemical Probing of PPT Architecture

The 3.0Å structure of a co-crystal of HIV-1 RT and a PPT-containing RNA/DNA hybrid [6] represented a major advance in our understanding of PPT architecture. Although RT was not positioned for RNase H-mediated cleavage at the biologically-relevant PPT/U3 junction, this structure nonetheless revealed anomalous hydrogen bonding between base pairs −9 and −15 (defining position −1 as the first g:C base pair upstream of the PPT/U3 junction (note that throughout the manuscript, RNA and DNA bases have been represented in lower and upper case, respectively). Collectively designated the “unzipped” portion, X-ray crystallography provided the first notion that PPT structural malleability could contribute to both RT binding and orientational bias. However, in the absence of high-resolution data for the RNA/DNA hybrid alone, whether anomalous base-pairing was a pre-existing feature of the PPT or the consequence of enzyme binding remains an unresolved issue.

**Figure 2.**
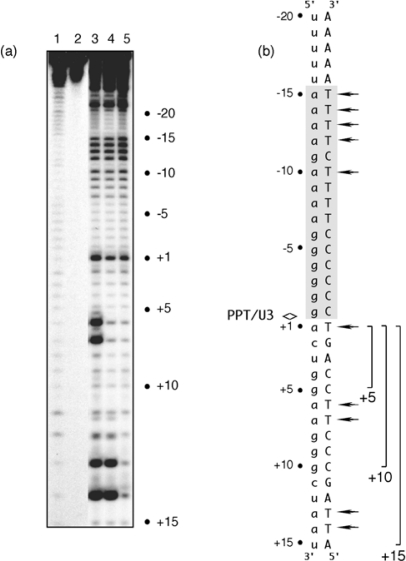
KMnO_4_ footprinting detects anomalous base pairing in the HIV-1 PPT DNA template in the absence of RT. (a), KMnO_4_ sensitivity of the minus-strand DNA template hybridized to PPT RNA primers containing a 5- (Lane 3), 10- (Lane 4) or 15-nt DNA extension (Lane 5), creating “staged” +5, +10 and +15 replication complexes. Lane 1, No KMnO_4_; Lane 2, No piperidine. As controls of KMnO_4_ sensitivity, +6T/+7T reactivity of the +5 replication complex is eliminated in a +10 complex, while +13T/+14T reactivity is eliminated in the +15 complex. (b), summary of KMnO_4_ footprinting data, indicating enhanced reactivity of +1T at the PPT/U3 junction and the distal (a) _4_:(T) _4_ tract. See [11] for additional details.

To address these two possibilities, we examined the sensitivity of PPT (−) DNA to KMnO_4_ oxidation. This probing strategy involves in-line attack of the C5-C6 double bond of unstacked and unpaired thymines in duplex DNA, and has been used extensively over the last two decades to examine DNA unwinding, promoter recognition and the structure of the transcription “bubble” [7–10]. In the absence of RT, we examined RNA/DNA hybrids whose PPT RNA had been chemically extended by 5, 10 or 15 deoxynucleotides as a means of mimicking “staged” polymerization complexes. Data shown in Figure 2 indicates that that template thymine −10 and those between −12 and −15 exhibited enhanced KMnO_4_ reactivity. Chemical probing of these artificially-generated replication complexes thus supported the earlier notion of enhanced PPT flexibility between regions −9 and −15, but suggested this might be an inherent feature of the RNA/DNA hybrid itself. An additional and unexpected finding was that all replication complexes exhibited enhanced KMnO_4_ sensitivity of template thymine +1 immediately adjacent to the PPT/U3 junction [11]. Using structural data to model the RNase H active site of HIV-1 RT directly over the PPT/U3 junction centered the “unzipped” portion ∼12 bp upstream where contact with critical motifs of the p66 thumb subdomain would be predicted [4–6]. The results of KMnO_4_ footprinting thus raised the possibility that cooperation between *two* structurally anomalous regions of the HIV-1 PPT might be required to promote correct positioning of RT for cleavage at the PPT/U3 junction. The notion that PPT malleability contributed to enzyme positioning was also supported by X-ray crystallography of a short portion of the HIV-1 PPT by Kopka *et al.* [12], which indicated altered stacking for the 5′-a-g-a- 3′ step located between positions −9 and −11.

Our chemical footprinting studies were complemented by examining PPT architecture via cleavage specificity of RNA/DNA hybrids containing strategically-positioned nucleoside analogs designed to alter duplex flexibility [13], base stacking [14] and backbone charge distribution [15]. Taken together, these studies reinforce the biological significance of altered geometry at both the PPT/U3 junction and the upstream unzipped region. More recently, hybrids whose RNA strand contained individual or multiple purine analog substitutions (e.g., purine, 2-aminopurine, diaminopurine, inosine, isoguanine) were examined in a comprehensive study of (+) strand primer selection specificity ^16^. Introducing analogs at positions +1, −2 and −4 adversely affected the specificity and efficiency of PPT/U3 cleavage, *i.e.*, cleavage at the junction was reduced, while abnormal cleavage in the immediate vicinity was observed. For PPT ribonucleotides +1a and −2g, the identity of the exocyclic group at position 2 of the purine ring was found to be critical. Specifically, at position −2g, a 2-NH_2_ group in the purine ring was essential, while the same chemical moiety at position +1a was not tolerated. In contrast, substrates modified at PPT position −4g required a carbonyl group at the purine ring 6-position for precise and efficient cleavage at the PPT/U3 junction.

**Figure 3.**
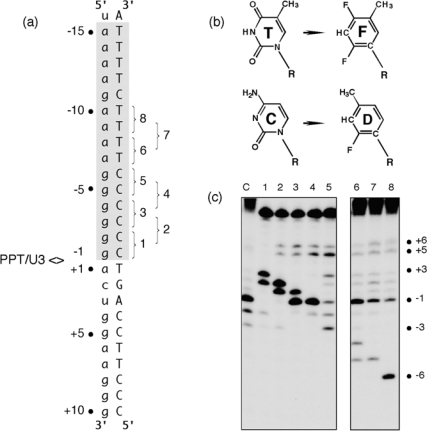
Pyrimidine isostere insertion into the PPT DNA template alters cleavage specificity. (a), Sequence of the HIV-1 PPT-containing RNA/DNA hybrid. T and C isosteres F and D, respectively, depicted in Panel (b) were added pairwise throughout the T_4_ and C_6_ tracts of the DNA template. PPT<>U3 denotes the scissile phosphodiester bond. Pairwise isostere insertions are numbered as follows: 1; −1D/−2D; 2, −2D/−3D; 3, −3D/−4D; 4, −4D/−5D; 5, −5D/−6D; 6, −7F/−8F; 7, −8F/−9F; 8, −9F/−10F. (c), Hydrolysis profiles of isostere-substituted PPT RNA/DNA hybrids. Lane notations correspond to the numbering system of Panel (a). Lane C, unmodified DNA. Adapted from [17].

The hypothesis that structural elements of HIV-1 RT contributed to the specificity of PPT recognition was also examined using RNA/DNA hybrids containing pyrimidine isosteres [17]. 2,4-difluoro-5-methylbenzene (F) and 2-fluoro-4-methylbenzene (D, Figure 3 (b)) are shape mimics of thymine and cytosine, respectively that, while sterically equivalent to their naturally occurring counterparts, do not participate in hydrogen bonding in the context of duplex nucleic acid [18,19]. To examine how the elimination of hydrogen bonding affected plus-strand primer processing, single or tandem F and D substitutions were introduced into the PPT DNA template (Figure 3 (a)). In apparent competition with normal PPT binding determinants, the RNase H catalytic center was consistently relocated to cleave 3–4 nt downstream from the site of analog substitution (Figure 3 (c)). This effect was observed on multiple substrates, although there appeared to be a direct relationship between the extent of RNA hydrolysis and the proximity of the analogs to the PPT/U3 junction. In many cases, anomalous cleavage occurred at the expense of normal PPT processing, while RT failed to cleave substrates containing F positioned opposite the PPT 5′ terminus.

Clearly, tandem insertion of pyrimidine isosteres was sufficient to relocate the RNase H catalytic center of HIV-1 RT to alternative sites of hydrolysis. Although the precise mechanism by which this unusual cleavage occurs requires further analysis, we propose that it reflected increased affinity of RT for nucleic acid at the sites of isostere insertion. A protein motif most likely to mediate this interaction is the RNase H primer grip, shown by structural studies to contact the DNA strand of an RNA/DNA hybrid ∼3–8 bp from the RNase H active site [6]. As potential binding sites within the PPT-containing hybrid are “sampled” by the retroviral enzyme, those providing a more flexible region for accommodating the RNase H primer grip (as might result from the loss of hydrogen bonding through isostere insertion) may be favored, leading to cleavage of the RNA strand 3–4 bp downstream from the site of analog substitution. Data in Figure 3 (c), Lane 5 also shows that the “tracking” pattern induced by isostere insertion was temporarily lost between the (g)_6_:(C) _6_ and adjacent (a)_4_:(T) _4_ tract, supporting our notion that a modular architecture provided by a:T and g:C tracts may be an important determinant of PPT cleavage specificity.

Surprisingly, while adjacent −1T, −2T -> F substitutions of the Ty3 PPT also eliminated cleavage at the PPT/U3 junction, this was accompanied by novel cleavage events significantly downstream at positions +10 and +11 [3]. The ∼12 bp spatial separation between the position of isostere insertion and novel cleavage sites suggested that the structural anomaly introduced by loss of hydrogen bonding was recognized by the thumb subdomain of the Ty3 enzyme as opposed to its RNase H primer grip.

## 3. Combining Chemical Probing with Mass Spectrometry

Selective 2′ hydroxyl acylation analyzed by primer extension, or SHAPE, has recently emerged as a rapid and facile method of examining RNA secondary structure by exploiting sensitivity of the ribose 2′ OH to acylation by N-methylisatoic anhydride (NMIA) in a constrained (paired) or flexible (unpaired) environment [20]. Ribose-specificity of NMIA thus allows all four bases of RNA to be probed with a single reagent. Applying SHAPE to the HIV-1 PPT RNA/DNA hybrid was hampered by our observation that the primer extension step was impaired at the (g)_6_ tract. Moreover, when examining analog-substituted PPTs, the size of the synthetic RNA/DNA hybrid (25–30bp) rendered primer extension impractical. However, since the acylation reaction of SHAPE produces a covalent adduct with characteristic ∼133-Da incremental mass, the extent and sites of NMIA modification of HIV-1 PPT were readily revealed by electrospray ionization-Fourier transform ion cyclotron resonance (ESI-FTICR) according to a strategy designated selective 2′ hydroxyl acylation analyzed by mass spectrometry, or SHAMS [21].

**Figure 4.**
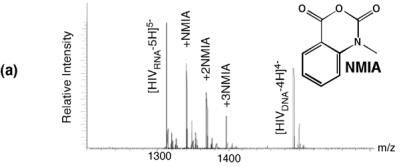

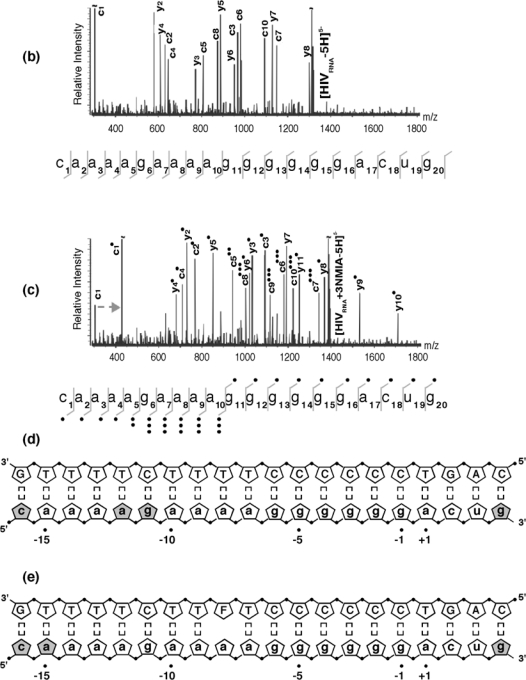
SHAMS – combining chemical modification and mass spectrometry to investigate PPT architecture. (a) Nano-ESI mass spectrum of the HIV-1 PPT RNA and DNA strands after treatment of the hybrid with a 10-fold excess of NMIA. Under these conditions, the majority of the RNA strand is unmodified, while the DNA strand, lacking a 2′ OH, is unaffected. (b), SORI-CID spectrum of the unmodified RNA substrate in the −5 charge state. For clarity, only the main sequence ions (c and y ions) are labeled using standard mass spectrometry notation. Gray lines through the sequence mark the cleavage positions corresponding to labeled fragments. (c) SORI-CID spectrum of the triply-modified RNA substrate in the −5 charge state. Only modified fragments are labeled for clarity. Due to modification of the RNA 2′ OH, the d-H_2_O series cannot undergo loss of H_2_O and therefore is observed as a c series. The gray arrow indicates the mass shift of 133.05 Da for one NMIA modification from the unmodified to the modified c_1_ ion. (•) indicates the number of NMIA modifications observed on the corresponding fragment, for example the c_5_•• ion corresponds to the ‘caaaa’ fragment with two modifications. (d), Schematic of the wild type HIV-1 PPT RNA/DNA hybrid. Gray pentagons indicate positions of NMIA sensitivity. (e) NMIA sensitivity of an HIV-1 PPT RNA/DNA hybrid whose DNA template contains a −8T -> dF substitution. Additional details of the SHAMS approach can be found in [21].

NMIA reactivity of the wild type HIV-1 PPT RNA/DNA hybrid is illustrated in Figure 4 (a). At a 10-fold excess of acylating agent, up to three adducts could be detected from their characteristic mass increment, together with a significant proportion of unmodified RNA. As predicted from the absence of a 2′ OH group, the PPT DNA strand was insensitive to NMIA modification. Together with earlier KMnO_4_ probing data [11], these results reinforced the hypothesis that discrete regions of the RNA/DNA hybrid deviated from canonical Watson-Crick base pairing, which received further support by examining the modified sites identified by tandem mass spectrometry. Gas-phase dissociation of nucleic acids produced characteristic backbone fragments that reflect the sequence of the precursor ion and reveal the position of covalent adducts [22]. Product ions spectra of unmodified and NMIA-modified PPT, which were produced by sustained off resonance irradiation-collision induced dissociation (SORI-CID) [23], are shown in Figure 4 (b) and (c), respectively, and the results are summarized in Figure 4 (d). The observed NMIA reactivity of terminal ribonucleotides reflected fraying of the duplex ends, which was consistent with previous NMR studies [24], while acylation of ribonucleotides −12a and −11g was interpreted as a consequence of their mispairing with template nucleotides −13T and −12T, respectively, consistent with the HIV-1 RT/PPT co-crystal structure [6]. However, NMIA reactivity did not support unpairing of ribonucleotide −13a, which had also been demonstrated crystallographically. In light of NMR observations (*see later*), we interpreted our NMIA probing data as reflecting a small population of the HIV-1 PPT with sufficient malleability to assume a configuration characterized by transient loss of base pairing. X-ray crystallography of an octanucleotide RNA/DNA hybrid derived from the HIV-1 PPT showed that anomalous stacking rendered the −12a/−11g/−10a step highly deformable, which may be sufficient to promote such a scenario. Alternatively, sensitivity of −12a/−11g to NMIA might be indicative of an alteration in sugar pucker from the C3′-*endo* to C2’-*endo* conformation, which has been suggested to mediate ligand recognition (which in this case would be the HIV-1 enzyme) and catalysis [25,26].

As described previously, our earlier work introducing the non-hydrogen-bonding thymine isostere F into the PPT DNA template showed that RNase H specificity was re-directed such that the PPT/U3 junction was no longer the preferential cleavage site [17]. A protein motif that might exploit weakened hydrogen bonding resulting from a T -> F substitution is the RNase H primer grip, which is proposed to impose the correct trajectory on the RNA strand of RNA/DNA hybrids for cleavage at the RNase H catalytic center [27–30]. Our SHAMS strategy was therefore extended to examine the consequences of thymine isostere insertion on PPT architecture with greater sensitivity, an example of which is shown in Figure 4 (e). On an RNA/DNA hybrid containing a −8T -> F substitution, the NMIA reactivity profile revealed only fraying of the terminal ribonucleotides, suggesting that (a) subtle changes in PPT architecture at the site of isostere insertion retained −8a ribose in an NMIA-insensitive configuration (e.g., through altering sugar pucker), and (b) changes in PPT architecture could be “sensed” at distal sites, suggesting that long range communication within this all-purine element may contribute to accommodating HIV-1 RT in either a polymerizing or hydrolyzing orientation.

## 4. High Resolution NMR Spectroscopy Analysis of the PPT

To further probe PPT RNA/DNA hybrids for pre-existing structural features, we have applied high resolution NMR techniques to analyze features of 20-mer RNA/DNA hybrid duplexes, representing the full wild-type (wt) Ty3 (5′–g-a-g-a-g-a-g-a-g-g-a-a-g-a-3′) and HIV-1 PPT sequences (5′-a-a-a-ag-a-a-a-a-g-g-g-g-g-g-3′) [24,31]. Proton and carbon chemical shifts, NOE and *J*-coupling data acquired for these hybrid duplexes indicated that regular Watson-Crick base-pairing and an A-form like helical conformation were adopted by both Ty3 and HIV-1 PPT. These overall structural features were not too surprising since r(purine):d(pyrimidine) hybrids had been previously reported to primarily adopt a global A-form geometry, although local conformational changes in ribose sugar pucker to a more B-form like geometry have been observed [32–34].

Despite the overall canonical nature of the hybrid duplexes, certain structural anomalies, manifested by ribose sugar pucker switching from C3′-endo to a mixed C3’/C2’-endo conformation, were detected in both the Ty3 and HIV-1 PPT sequences. Most interestingly a sugar pucker switch from C3’-endo to mixed C3’/C2’-endo was observed at ribonucleotide +1 of the Ty3 PPT [31], which was consistent with earlier KMnO_4_ probing data showing sensitivity at template thymine +1 of the HIV-1 PPT and postulated to contribute an additional ‘distortable’ feature to ensure alignment of the RNase H active site for accurate cleavage. In addition, more subtle line width analysis of the imino proton resonances from the HIV-1 PPT revealed broader line widths for −1g, −7T, and −11g, which would be consistent with the PPT being more prone to distortion in the regions of transition between the two upstream a:T tracts as well as between the central a:T and adjacent g:C tract. Thus, while the imino proton NMR data did not indicate either mispairing or unpairing of bases in the HIV-1 PPT, line width analysis corroborates KMnO_4_ data with the broadening of the imino resonances indicative of a change in the lifetime of base-pair opening and a potentially greater solvent accessibility for these bases [35].

As with our other chemical and biophysical approaches, NMR analysis of the Ty3 HIV-1 PPTs was further advanced by examining RNA/DNA hybrids containing either single base-pair changes or introducing the thymine isostere F into the DNA template to probe for subtle structural perturbations. Using NMR, we sought to uncover structural perturbations resulting from single-site F substitutions that could be correlated with biochemical data that demonstrated a decreased fidelity of RNase H processing for single F substitutions [17]. Of the substituted DNA strands, significant alterations in the ribose sugar puckering patterns were observed with both -9F - and -11F-substituted Ty3 PPTs, with both resulting in an internal shift in the RNA strand to a more B-form like geometry. What was especially intriguing about this phenomenon was our finding that a mutation in the DNA base induced structural changes downstream in the ribose sugars of the RNA strand. This unanticipated observation clearly demonstrated a long range structural coupling in the retroviral and retrotransposon PPTs. In the most dramatic case, a -11F substitution of the Ty3 PPT induced a periodic propagation of altered sugar puckering in every other ribose to the 3′ end of the helix and may be a result of multiple 5′-a•g•a-3′ symmetric steps within this RNA/DNA hybrid. In contrast, long range structural effects were more limited in the HIV-1 PPT, *i.e*., only the HIV +1T -> F-substituted hybrid exhibited a long range ribose sugar pucker switch at ribonucleotide −10a. Conceivably, asymmetry between the g:C tract and two a:T tracts may lessen the propagation of structural effects along the HIV-1 PPT.

Interestingly, PPT hybrid sequences containing single base alterations of the DNA template (e.g. Ty3 -8C -> T and HIV-1 -3C -> T) exhibited structural effects similar to the F-substituted hybrids, suggesting that structural effects observed in the latter samples were not due solely to incorporation of a fluorinated base. While less pronounced as F-substituted samples, these Ty3 PPT mutants showed a similar propagation of ribose sugar pucker switches towards the 3′ end of the RNA strand. On the other hand, the HIV-1 -3C -> T substituted hybrid exhibited ribose sugar puckering consistent with both HIV -8T -> F and HIV WT, where only end-fraying was observed. While the PPT g:C tract is highly conserved across lentiviruses, and mutations tend to decrease the fidelity of RT-mediated processing, no striking differences in the global structure of the HIV-1 -3C -> T mutant hybrid could be detected. Overall, results from dF-substituted RNA/DNA hybrids suggested greater helical flexibility for the Ty3 PPT. While this may simply reflect differences in sequences as described above, this was also unexpected in light of the mispaired and unpaired bases observed in the HIV-1 PPT•RT crystal structure. Nonetheless, the inherent PPT flexibility evidenced by the T -> dF substitutions suggests that upon RT binding, structural perturbations throughout the sequence promote contacts with the appropriate protein motifs, leading to productive binding and cleavage at the correct site.

To determine if the mispaired and unpaired bases observed in the HIV-1 PPT•RT crystal structure were induced by the binding of RT, an attempt was made to monitor the effect of RT binding on the imino proton spectra of the HIV-1 PPT by solution state NMR. In this experiment, while general broadening of all imino proton signals was observed, no evidence for a G•T mismatch was found. Nonetheless, more significant signal broadening and some minor chemical shift perturbations were detected for resonances associated with bases in the 5′ (a)_4_:(T) _4_ tract and the PPT/U3 junction where RT is predicted to make direct contacts.

## 5. Single Molecule Spectroscopy and RT Orientational Dynamics

**Figure 5.**
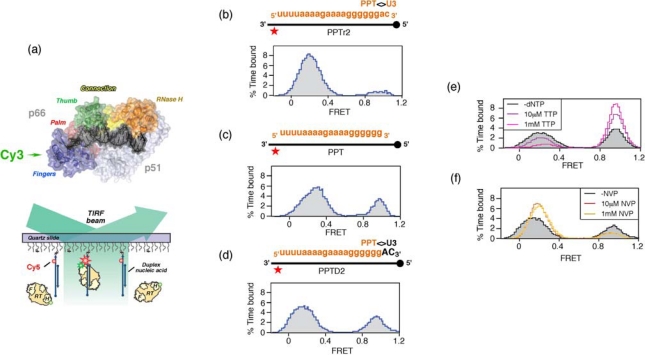
Examining RT orientational dynamics by single molecule spectroscopy. (a), *Upper,* HIV-1 RT is site-specifically labeled with Cy3 (green) at its C-terminal RNase H domain. *Lower,* Cy3-labeled RT interacts with surface-immobilized Cy5-labeled DNA, and fluorescence of individual substrates is followed by total-internal-reflection fluorescence (TIRF) microscopy with alternating laser excitations at 532 and 635nm. Panels (b) – (d), Alternative RT orientations on the PPT. FRET histograms derived from Cy3 RNase H-labeled RT incubated with RNA/DNA hybrids whose PPT RNA primer was (b), extended at its 3′ terminus with two ribonucleotides (PPTr2), (c), lacking an extension (PPT) or (d), extended by two deoxynucleotides (PPTD2). (e) Ternary complex formation promotes enzyme binding to the primer terminus in a polymerization orientation. Histogram of RT bound to PPT:dd2 hybrid (filled, grey) in the presence of 10 μM (purple trace) and 1mM TTP (cyan trace) (f), NNRTI binding induces RT binding to the PPT 3′ terminus in an RNase H orientation. Histograms of RT bound to PPT:d2 substrates in the absence (filled grey trace) presence of 10 μM (red trace) and 100 μM nevirapine (NVP) (orange trace). See [36] for additional details.

Since plus-strand DNA synthesis requires the PPT 3′ terminus to be accurately recognized by catalytic centers located at either end of HIV-1 RT, the mechanism by which enzyme orientation is controlled becomes an issue. As these processes are less amenable to ensemble-based strategies, we elected to examine the interaction of HIV-1 RT with structurally-diverse nucleic acids by single molecule spectroscopy [36,37]. Our strategy is schematically summarized in Figure 5 (a), and required positioning a fluorescent energy resonance transfer (FRET) donor (Cy3) on the N- or C-terminus of the p66 RT subunit, and an acceptor dye (Cy5) on surface-immobilized nucleic acid duplexes. Since the p66 termini of the p66/p51 RT heterodimer are ∼100Å apart, enzyme orientation could be inferred from the strength of the FRET signal Validating the biological significance of our single molecule approach required demonstrating that both variants of Cy3-labeled RT retained the capacity to extend the primer of an immobilized DNA duplex [36].

Consistent with biochemical studies, HIV-1 RT positioned its DNA polymerase active site over the primer 3′ OH of duplex DNA [4,5], and this orientation persisted when the DNA template was replaced with an RNA of equivalent sequence. However, hybridizing an RNA *primer* of equivalent length and sequence to the DNA template favored the opposite orientation, *i.e*., the RNase H domain was now over the primer 3′ terminus. We next examined chimeric RNA-DNA primers whose RNA content progressively increased from the 5′ terminus, demonstrating that as few as two 5′ ribonucleotides could initiate enzyme re-orientation, and that the process was virtually complete when the primer contained five 5′ ribonucleotides. The co-crystal of HIV-1 RT and a PPT-containing RNA/DNA hybrid indicates that Glu89, Gln91, Cys280 and Ala284 of the p66 subunit contact the ribose 2′-OH at several positions near the RNA 5′ terminus. These contacts would be absent with duplex DNA [4,5], raising the possibility that enhanced hydrogen bonding with the RNA strand stabilized an orientation where its 5′ terminus is accommodated in the DNA polymerase catalytic center, *i.e*., the primer is effectively recognized as the template.

Although this hypothesis requires further experimentation, the observation that HIV-1 RT adopted alternate orientations on nucleic acid duplexes in the absence of accessory host or viral proteins provided a first clue to mechanisms of PPT selection. In Figure 5 (b) – (d), we examined enzyme orientation on PPT-containing RNA/DNA hybrids designed to mimic selection of the primer 3′ terminus and its subsequent extension into plus-strand DNA. On an RNA primer extended by two ribonucleotides beyond the PPT/U3 cleavage junction (PPTr2), the FRET histogram of Figure 5 (b) indicated a predominant enzyme orientation that positioned the RNase H domain for cleavage at this junction. Removing these two ribonucleotides to mimic liberating the PPT 3′ terminus for initiation of plus-strand DNA had the consequence that a significant fraction of enzyme assumed a polymerization-competent orientation on the all-RNA primer (Figure 5 (c)). Finally, extending the PPT by two deoxynucleotides (PPTD2), mimicking initiation of plus-strand DNA synthesis, promoted both enzyme orientations (Figure 5 (d)). The ability of the PPT to support alternate enzyme orientations provided a mechanism whereby the plus-strand primer would be created, extended and precisely removed from nascent DNA. Although an RNase H orientation would be required to remove the RNA primer from plus-strand DNA, an interesting issue is the extent of DNA-dependent DNA synthesis from the PPT 3′ terminus that is required to induce exclusively the polymerization orientation.

To investigate how structural features of the HIV-1 PPT supported alternate enzyme orientations, the interaction of RT with a hybrid containing a chain-terminated primer (PPTD2) was examined in the presence of the incoming dNTP, which would establish a stable ternary complex [4]. As would be predicted, Figure 5 (e) illustrates that these conditions strongly favor the polymerization-competent binding mode. Conversely, nevirapine, a nonnucleoside RT inhibitor (NNRTI) increased the frequency with which enzyme bound the primer terminus in the RNase H-competent mode (Figure 5 (f)). NNRTI-induced re-orientation of HIV-1 RT in a manner incompatible with polymerization may explain observations that this class of drug was found to preferentially inhibited initiation of HIV-1 plus-strand DNA synthesis [38].

Structurally, the incoming dNTP and nevirapine should exert opposite effects on RT conformation within and close to the DNA polymerase catalytic center. While the former will induce tightening of the p66 fingers and thumb subdomains around nucleic acid in a ternary complex [4], nevirapine is located within a hydrophobic pocket at the base of the p66 thumb [39] and is predicted to loosens its grip on the duplex. Consistent with the concept that loosening contact with duplex DNA was a consequence of NNRTI binding, nevirapine was later demonstrated to enhance the ability of HIV-1 RT to “slide” over duplex DNA [37].

## 6. Interaction of Small Molecules with the HIV-1 PPT

**Figure 6.**
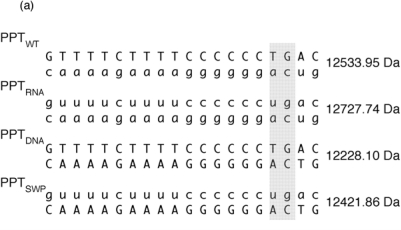

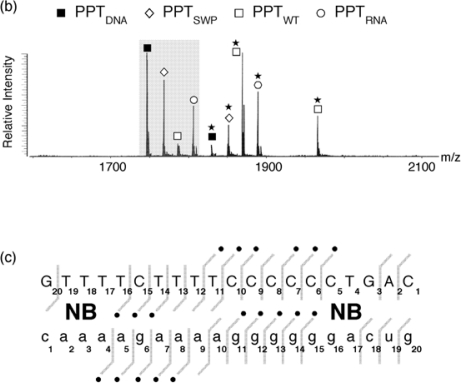
Investigating aminoglycoside binding to the HIV-1 PPT by mass spectrometry. (a) Sequence and masses of the wild type HIV-1 PPT (PPT_WT_), an all-DNA version (PPT_DNA_,), an all RNA version (PPT_RNA_), and a hybrid whose DNA and RNA sequences were interchanged (PPT_SWP_); (b) ESI-FTICR spectrum of an equimolar mixture of PPT variants in the presence of neomycin B (NB). Mass signatures for unliganded duplexes are illustrated in the grey filled portion of the spectrum. In the presence of neomycin B, mass increments for each duplex suggest that PPT_RNA_, PPT_DNA_ and PPT_SWP_ bind one equivalent of ligand, while two equivalents bind to PPT_WT_. (c) Determination of the neomycin B binding sites on PPT_WT_ by tandem mass spectrometry. The ion series observed for the DNA and RNA strands constituting the substrate are reported on the respective sequences to highlight nucleotides prevented from undergoing fragmentation in the presence of ligand [48]. The lines mark the phosphodiester bonds cleaved by gas-phase dissociation. Conversely, the absence of phosphodiester bond cleavage indicates protection by bound neomycin B. See [44] for additional details.

Sensitivity of template nucleotide +1T to KMnO_4_ oxidation [11] raised the possibility that altered nucleic acid geometry might augment “docking” of the scissile phosphodiester bond at the PPT/U3 junction into the RNase H catalytic center. This notion was in part supported by our SHAMS analysis of a PPT RNA/DNA hybrid containing the pyrimidine isostere dF in place of template nucleotide +1T [21]. Figure 4 (e) demonstrates that while base pairing is lost, +1a ribose remained NMIA-insensitive, suggesting a unique environment at the junction with significant stacking rigidity. To further investigate the notion of structure-specific recognition, we exploited the ability of the aminoglycoside neomycin B to interact with nucleic acid motifs that disrupt regular double-helical patterns, including non-canonical pairs, platforms, bulges, bends, and other anomalies [40]. These peculiar features can provide unique H-bonding opportunities for the polar groups of aminoglycosides, which are placed in optimal positions by the flexible 2-deoxystreptamine scaffold [40]. Electrostatic interactions between protonated amino groups on the aminoglycoside and highly electronegative patches on the nucleic acid surface further stabilize neomycin B binding. The application of mass spectrometry to study PPT/ligand interactions is exemplified in Figure 6. In addition to determining the stoichiometry and site(s) of ligand binding, mass spectrometry has the additional advantage of permitting multiplexing, *i.e*., simultaneously examining ligand binding to multiple PPT-containing substrates.

Figure 6 (a) provides the sequence and unique molecular mass of the wild type PPT (PPT_WT_), all DNA (PPT_DNA_), and all-RNA variants (PPT_RNA_), and a third variant whose RNA and DNA strands were interchanged (PPT_SWP_). In the presence of neomycin B, ESI-FTICR mass spectrometry identified the unliganded nucleic acid duplexes, in addition to their complexes containing one equivalent of neomycin B (Figure 6 (b)). The exception to this was PPT_WT_, which Figure 6 (b) indicates bound two equivalents of the aminoglycoside. As with our SHAMS approach, tandem mass spectrometry allowed us to determine the site of neomycin B binding from the fragmentation pattern afforded by SORI-CID. Data in Figure 6 (c) indicate binding sites in the immediate vicinity of the PPT/U3 junction, and approximately 13–14 bp upstream, in the distal a:T tract.

**Figure 7.**
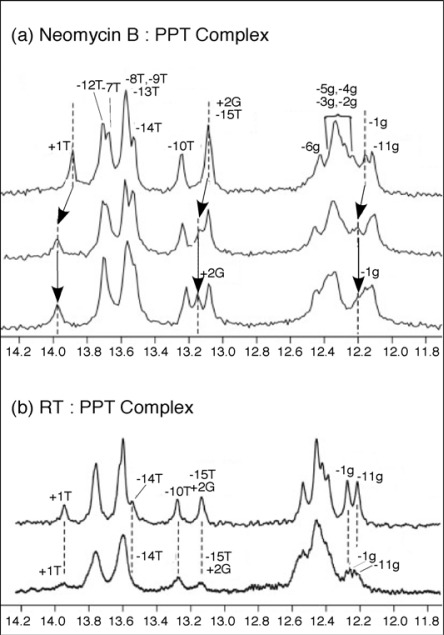
One-dimensional water flip-back watergate ^1^H NMR spectra of the imino region of the PPT_wt_ duplex after titration with neomycin B (Panel (a)), or HIV-1 RT (Panel b)). In (a), 1D ^1^H NMR spectra were obtained in the absence (*upper*) and presence of 1.0 equivalents (*middle*) and 2.0 equivalents (*lower*) of neomycin B. Imino resonances for +2G, +1T and −1g where chemical shift changes can be tracked are highlighted. Dotted lines and arrows indicate the shift in position of the resonances. (b) 1D ^1^H NMR spectra for PPT_wt_ in the absence (*upper*) and presence of 1.0 equivalent (*lower*) of p66/p51 HIV-1 RT. Imino protons were assigned using 2D NOESY experiments; assignments are listed above each peak. Adapted from [41].

To substantiate the results of our mass spectrometry strategy and obtain complementary structural information, solution NMR was employed to map neomycin B binding to the wild-type PPT (Figure 7). Addition of one equivalent of neomycin B to PPT_wt_, resulted in a shift in the imino proton signals for −1g, +1T, and +2G, consistent with selective binding at the PPT-U3 junction (Figure 7(a), *middle*). After the primary site was saturated, addition of a second neomycin B equivalent did not introduce any distinct shifts in the imino proton spectrum. Instead, general broadening of all signals suggested weaker, non-specific binding interaction(s) between ligand and the PPT substrate (Figure 7 (a), *lower*). While the NMR data did not allow mapping of this secondary site(s), the observation of consecutive binding events in the NMR titrations was consistent with high and low affinity sites revealed by mass spectrometry. Moreover, NMR data indicated that changes in the imino proton spectrum of the PPT in the presence of HIV-1 RT were consistent with the retroviral enzyme making contact with both ends of the RNA/DNA hybrid (Figure 7 (b), *lower*) [41].

Taken together, two features of the PPT_WT_/neomycin B complexes were apparent from the combined mass spectrometry and NMR data. Firstly, it supported our original hypothesis that anomalous nucleic acid geometry most likely contributes to positioning the RNA strand in the RNase H active site for precise cleavage at the PPT/U3 junction. Secondly, the 13–14 bp separation of the two neomycin B binding sites corresponds closely to the length of nucleic acid between the RNase H catalytic center and the thumb subdomain of the p66 RT subunit, the latter of which houses a “translocation motif” implicated in minor groove tracking [42,43]. Based on crystallographic data demonstrating “unzipping” of the PPT [30], we have proposed that a unique geometry located at either extremity of the PPT-containing duplex is recognized by structural motifs of the HIV-1 enzyme to facilitate positioning and accurate RNase H-mediated cleavage at its 3′ terminus.

Finally, mass spectrometry and NMR were also used to characterize the interaction of neomycin B with the Ty3 PPT. This study [44] revealed equivalent aminoglycoside binding sites, suggesting that, despite a significant difference in PPT sequence, the Ty3 RT thumb subdomain and RNase H domain also appear to recognize structural distortions located at either extremity of the RNA/DNA hybrid. To determine whether these sites might be structurally coupled, hybrid duplexes lacking either one or both were examined. In one construct, the 5′-end of the RNA strand was modified to a g•a polymer (PPT_distal_), while in the second, the 3′-end of the RNA was similarly modified to remove PPT/U3 binding site (PPT_prox_). Lastly, both sites were simultaneously eliminated by preparing a continuous g•a sequence (PPT_pol_). Should each site bind neomycin B independently, the affinity for PPT_distal_ and PPT_prox_ would be similar to the corresponding site in the wild type RNA/DNA hybrid. Conversely, if the binding sites were structurally coupled, affinities for the remaining sites might be weaker than the wild type, or eliminated. For PPT_pol_, where both sites were removed, only nonspecific binding was expected. Mass spectrometry and NMR analysis illustrated that the mutant PPT constructs displayed altered aminoglycoside binding stoichiometry and affinity, with binding retained only at the analogous wild-type binding positions. Binding at the remaining wild-type sites, however, showed altered kinetics on the NMR time-scale. The apparent affinity for the remaining site of the mutants was also weaker, with two equivalents of neomycin B needed to achieve saturation. Collectively these results reinforced the notion of structural coupling, *i.e*., removing the proximal or distal regions of the PPT not only repressed binding of a second neomycin B equivalent, but also altered its affinity and binding kinetics for the remaining site.

## 7. Conclusions

In addition to providing new insights into the molecular interplay between RT and its cognate PPT, the studies reviewed here emphasize the notion that a comprehensive analysis of nucleic acid structure, and its effects on associated protein/nucleic acid interactions, requires combining a variety of biochemical and biophysical techniques capable of providing complementary information. This is of particular importance as new strategies based on SHAPE and related techniques emerge with the capacity to rapidly probe the structure of large RNA molecules [20,45] and, recently, the entire HIV-1 genome [46]. While such approaches can provide insight into key secondary structure elements, important tertiary interactions can be overlooked. Complementary to SHAPE, tertiary structure information can be provided by small angle X-ray scattering, which has experienced excellent advances, and by recently-developed MS3D approaches, which combine structural probing via bifunctional crosslinking agents with mass spectrometric detection to identify long-range tertiary contacts [47]. As 3D models are developed and refined, they will lend themselves to dissection and high resolution analysis by NMR spectroscopy and X-ray crystallography, possibly making use of nucleoside analogs similar to those highlighted here. The techniques we have briefly reviewed, while originally confined to specialized groups, are now finding everyday application in most laboratories. In combination, these approaches promise to provide deeper and richer structural insight on nucleoprotein complexes with unprecedented speed and accuracy.
